# Hepatitis C Virus and Cellular Stress Response: Implications to Molecular Pathogenesis of Liver Diseases

**DOI:** 10.3390/v4102251

**Published:** 2012-10-19

**Authors:** Po-Yuan Ke, Steve S.-L. Chen

**Affiliations:** 1 Department of Biochemistry and Molecular Biology, College of Medicine, Chang Gung University, Taoyuan 33371, Taiwan, Republic of China; Email: pyke0324@ibms.sinica.edu.tw (P.-Y.K.);; 2 Institute of Biomedical Sciences, Academia Sinica, Taipei 11529, Taiwan, Republic of China

**Keywords:** HCV, host factor, cellular response, autophagy, ER stress, unfolded protein response, apoptosis, DNA damage, cell cycle arrest, liver diseases

## Abstract

Infection with hepatitis C virus (HCV) is a leading risk factor for chronic liver disease progression, including steatosis, cirrhosis, and hepatocellular carcinoma. With approximately 3% of the human population infected worldwide, HCV infection remains a global public health challenge. The efficacy of current therapy is still limited in many patients infected with HCV, thus a greater understanding of pathogenesis in HCV infection is desperately needed. Emerging lines of evidence indicate that HCV triggers a wide range of cellular stress responses, including cell cycle arrest, apoptosis, endoplasmic reticulum (ER) stress/unfolded protein response (UPR), and autophagy. Also, recent studies suggest that these HCV-induced cellular responses may contribute to chronic liver diseases by modulating cell proliferation, altering lipid metabolism, and potentiating oncogenic pathways. However, the molecular mechanism underlying HCV infection in the pathogenesis of chronic liver diseases still remains to be determined. Here, we review the known stress response activation in HCV infection *in vitro* and *in vivo*, and also explore the possible relationship of a variety of cellular responses with the pathogenicity of HCV-associated diseases. Comprehensive knowledge of HCV-mediated disease progression shall shed new insights into the discovery of novel therapeutic targets and the development of new intervention strategy.

## 1. Introduction

**Figure 1:**
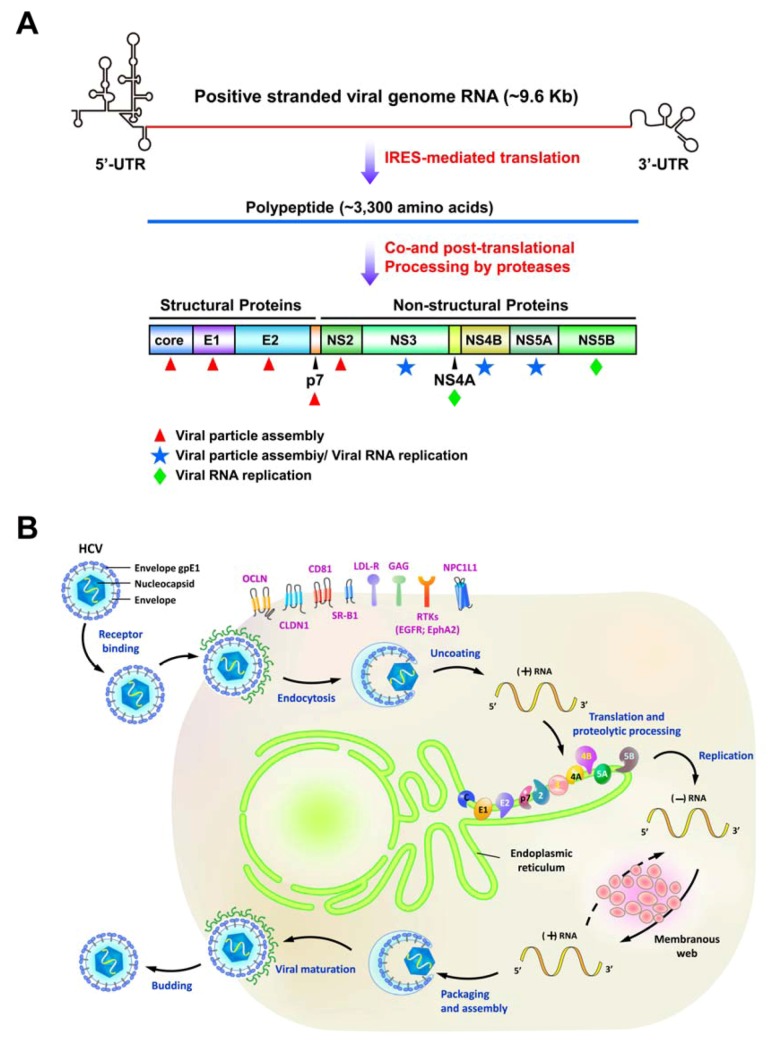
The genomic organization and life cycle of hepatitis C virus (HCV). (A) Schematic diagram of the HCV genome. The positive-stranded genome RNA of HCV is of approximately 9.6 Kb and is flanked by the 5´- and 3´untranslated regions (UTR). The coding sequence of HCV viral RNA encodes a single polypeptide through internal ribosome entry site (IRES)-mediated translation. The nascent translated polypeptide is subsequently processed by a combination of cellular and viral proteases to mature into structural proteins (core, E1, E2, and p7) and nonstructural proteins (NS2, NS3, NS4A, NS4B, NS5A, and NS5B). Core, E1, and E2 constitute the components of viral particle (red asterisks) whereas NS4A and NS5B specifically function in the replication of viral RNA. NS2 and p7 are involved in the assembly of viral particles. NS3, NS4B, and NS5A have its dual role in both viral replication and assembly. (B) Schematic representation of the HCV viral life cycle. The viral particles associated with lipoproteins enter into host cells via (co)receptor binding and clathrin-mediated endocytosis. The known entry (co)receptors, tetraspanin CD81, the scavenger receptor class B member I (SR-BI), Claudin 1 (CLDN1), Occludin (OCLN), glycosaminoglycans (GAG), the low-density lipoprotein receptor (LDLR), epidermal growth factor receptor (EGFR), ephrin receptor A2 (EphA2), and Niemann-Pick C1-like L1 (NPC1L1) are indicated. After uncoating process, the positive-stranded viral RNA is released, translated, and processed into different viral proteins. The NS viral proteins mediate the replication of positive-stranded viral RNA within a membranous structure, called membranous web. The infectious viral particles containing the newly-synthesized viral RNA and structural proteins are assembled and egressed via the secretory pathway.

Hepatitis C virus (HCV) is an enveloped, positive-sense, single-stranded RNA virus classified within Hepacivirus genus of the Flaviviridae family [1]. Its viral genome is about 9.6 Kb in length and is uncapped, which is flanked by untranslated regions (UTRs) at its 5´ and 3´ ends [2] (Figure 1A). HCV mainly targets hepatocytes and its infection is mediated by several entry cofactors located on the cell surface, including the tetraspanin CD81, the scavenger receptor class B member I (SR-BI), Claudin 1 (CLDN1), and Occludin (OCLN) [3,4,5,6] (Figure 1B). Associated with lipoproteins as a complex, the viral particle attaches to the glycosaminoglycans (GAG) and the low-density lipoprotein receptor (LDLR), and then interacts with CD81 and SR-BI [7]. Subsequent re-locating to the tight junction containing CLDN1 and OCLN, the viral particle becomes internalized via the pH-dependent, clathrin-mediated endocytosis. Despite of these well-known entry (co)receptors of HCV infection, the epidermal growth factor receptor (EGFR) and ephrin receptor A2 (EphA2) have been identified as new (co)factors for HCV entry by promoting CD81-CLDN1 association and viral glycoprotein-dependent membrane fusion via their receptor tyrosine kinase (RTK) activities [8]. Very recently, Sainz *et al.* also reported that the Niemann-Pick C1-like L1 (NPC1L1) cholesterol uptake receptor mediates HCV entry in a cholesterol-dependent manner [9]. After internalization by the clathrin-mediated endocytic process, the envelope glycoproteins of viral particles then fuse with the endosomal membrane to release viral genome into the cytoplasm. The viral RNA encodes a single polypeptide of about 3,000 amino acids (a.a.) that is cleaved by cellular and viral proteases into 10 different proteins [2] (Figure 1A). The four structural proteins core, E1, E2, and p7 constitute the viral particle [1,2], whereas six nonstructural protein (NS) proteins NS2, NS3, NS4A, NS4B, NS5A, and NS5B participate in the replication of viral RNA and the assembly of viral particle [2]. Recent studies indicate that lipid droplets (LDs) play a key role in HCV life cycle [10,11,12,13]. The core protein directly localizes onto the surface of LDs and then recruits other NS proteins to the LDs [12]. Interruption of the association between core and LDs or interference with NS5A and core-coated LDs decreased the infectivity of viral particle, indicating that LDs function in the assembly of infectious HCV [10,11,12,13].

**Figure 2:**
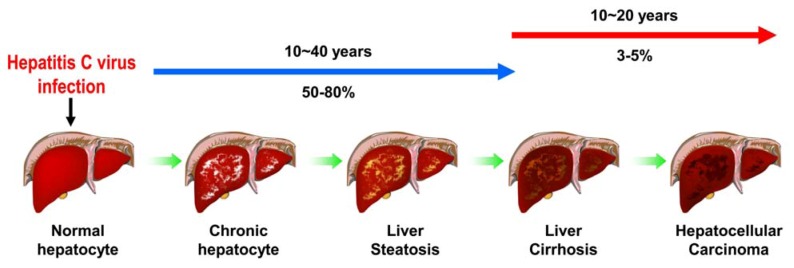
Pathogenesis of HCV-associated liver diseases. Approximately 3% of the population has been infected with HCV worldwide. In the majority (about 50-80%) of infected individuals it becomes a persistent-infection and the chronic-infected hepatocytes in liver progressively develop into liver steatosis, and liver cirrhosis. Ultimately, the chronic infection leads to hepatocellular carcinoma formation in nearly 3-5% of viral-infected patients.

HCV infection is a major challenge of public health, with approximately 3% of population infected worldwide [1]. The majority (50-80%) of infected individuals becomes chronic hepatitis which progressively develops into hepatosteatosis, liver fibrosis, liver cirrhosis and ultimately to hepatocellular carcinoma [14] (Figure 2). Current standard of treatment against HCV infection comprises pegylated interferon-α and ribavirin [15]. However, the severe side effects and different efficacy in treating infections with various genotypes restrict the success rate of this combined therapy [15]. Recently, several studies have shown that HCV possesses an ability to activate various cellular responses, including endoplasmic reticulum (ER) stress/unfolded protein response (UPR), autophagy, apoptosis, and cell cycle arrest. These cellular responses triggered by virus infection have been implicated to be exploited by host cells to counteract viral infection or by virus to promote its growth, and thus maintaining the homeostasis between HCV and host cells. Also, emerging evidence suggests that these cellular responses may participate in the pathogenesis of HCV-associated liver diseases, such as by altering lipid metabolism, interfering with cell growth and/or proliferation, and activating oncogenic signal pathway. The aim of this review is to provide updated information on the relationship between HCV-induced cellular responses and the chronic liver diseases associated with viral infection. Hence, we overview recent findings in this respect, in particular, on ER stress and UPR, autophagy, apoptosis, and cell cycle arrest and DNA damage. We also present the current understanding of the HCV-induced cellular responses and their related mechanisms and the physiological significance of these host cellular responses in the pathogenesis of chronic liver diseases. At last, we discuss the perspective and implications of the related research in the clinical therapy and the future development of new antiviral strategy.

## 2. Various Cellular Responses Activated by HCV

### 2.1. ER stress and UPR

**Figure 3:**
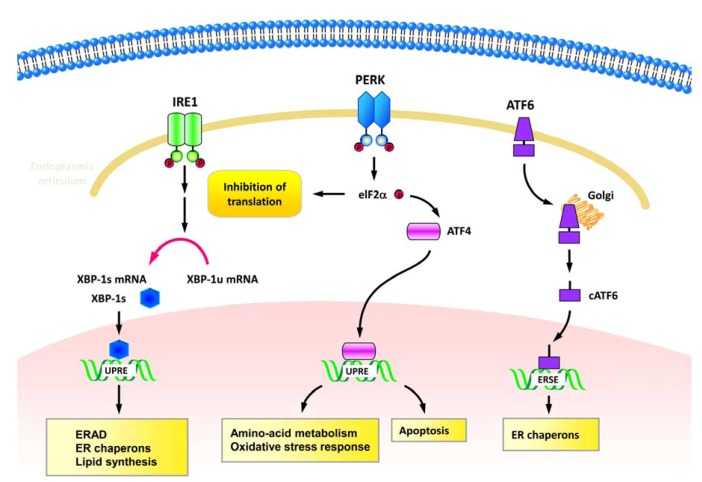
Signaling transduction of UPR. Three major signaling transducers of UPR, including inositol-requiring protein-1 (IRE1), activating transcription factor-6 (ATF6), and protein kinase (PKR)-like ER kinase (PERK) are indicated. Left column: Upon sensing accumulation of unfolded proteins, IRE1 oligomerizes and processes autophosphorylation, leading to X-box protein-1 (XBP1) mRNA splicing. The spliced XBP-1 (XBP-1s) subsequently transactivates gene expressions of chaperones, lipogenic genes, and ER-associated degradation (ERAD). Middle column: In response to ER stress, PERK also undergoes oligomerization and autophosphorylation processes. The activated PERK then transduces the signaling to activate eukaryotic translation initiation factor 2α (eIF2α) via phosphorylation, resulting in suppression of global protein translation and activations of UPR genes that function in amino acid transporter, oxidative response, and apoptosis. Right column: When cells facing ER stress, the precursor form of ATF6 is first translocated from the ER to Golgi apparatus and processed into the cleaved form of ATF6 (cATF6). Then the cATF6 is shuttled into nucleus to transactivate UPR target genes, such as ER chaperons.

Replication of HCV viral genome occurs at the replication complex which is organized within the ER-associated membranous structures, the so called membranous webs [16,17,18] (Figure 1B). HCV NS4B has been shown to trigger the formation of membranous web by inducing the rearrangement of ER-derived membranes [16,17] (Table 1). In addition, HCV utilizes the ER-membranous compartment as the site of envelope protein biogenesis and viral particle assembly [19,20]. Thus, it is believed that HCV infection may induce ER stress and interfere with the function of ER in host cells. The ER is a cellular organelle for folding and modification of membrane-bound and secreted proteins. The imbalance between a large amount of proteins accumulated in the ER and the limited folding capacity of ER machinery induces a stress response, known as ER stress. Upon sensing ER stress, cells activate a unique intracellular signaling pathway, the UPR [21]. Three classes of signal transducers of UPR have been defined, *i.e.,* the inositol-requiring protein 1 (IRE1), activating transcription factor-6 (ATF6), and protein kinase (PKR)-like ER kinase (PERK) signal pathways, and each arm of signal pathways presents a distinct role in UPR activation [21] (Figure 3). In response to unfolded proteins, IRE1 oligomerizes on the ER membrane and is then activated by trans-autophosphorylation of the juxtaposed kinase domain [22,23,24] (Figure 3). The activated IRE1 signaling triggers the endonucleolytic cleavage of X-box protein-1 (XBP1) mRNA, resulting in the conversion of the unspliced XBP1 (XBP-1u) to the spliced form of XBP-1 (XBP-1s) [25,26] (Figure 3). The XBP-1s protein encoded by the XBP-1s mRNA is more stable and functions as a potent transcriptional activator of UPR genes that contain the unfolded protein responsive elements (UPREs), including chaperones, lipogenic genes, and ER-associated degradation (ERAD)-related genes [27,28] (Figure 3). Under the condition of ER stress, ATF6 is translocated from the ER to Golgi apparatus, where the N-terminus is cleaved by Golgi-resident proteases [29,30,31]. After proteolytic processing, the cleaved form of ATF6 (cATF6) with the cytosolic DNA binding portion is released from membrane and then shuttled into nucleus to transactivate UPR target genes with the ER stress responsive elements (ERSEs) [32,33] (Figure 3). The third signaling transduction module of UPR is initiated by oligomerization and autophosphorylation of PERK [34,35] (Figure 3). The activated PERK subsequently phosphorylates eukaryotic translation initiation factor 2α (eIF2α) and results in suppression of global protein translation and activations of UPR genes involved in amino acid transporter, oxidative response, and apoptotic cell death [36,37,38,39] (Figure 3). Activation of these three modules of UPR signaling leads to a coordinative response which reduces the unfolded proteins that enter into ER, downregulates protein translation and translocation, and increases the capacity of the ER to tolerate unfolded proteins [21]. Finally, the UPR improves the efficiency of protein folding, removes misfolded proteins through the ERAD system, or triggers cell death [21,40] (Figure 3).

Viral infection often induces ER stress and activates UPR, including herpes simplex virus type 1, human cytomegalovirus, and Epstein-Barr virus. In the case of HCV, Tong *et al*. first showed that HCV NS4B protein interacts with ATF6 protein through its N-terminal region [41] (Table 1), implying that HCV gene expression may modulate ER stress and UPR. In line with this study, replication of HCV subgenomic replicon which contains only the genome fragment of NS3 to NS5B in human hepatoma Huh7 cells leads to the cleavage of ATF6 and transcriptional activation of UPR chaperon protein, 78-kDa glucose regulated protein (GRP78) [42] (Table 1). Further studies demonstrated that HCV NS4B can induce ATF6 and IRE1-XBP1 splicing, two signal transduction modules of UPR through stimulating the production of reactive oxygen species (ROS) and perturbing calcium homeostasis [43,44] (Table 1). Activation of UPR by HCV NS4B was shown to interfere with the activity of ERAD, and enhance the nuclear factor kappa-light-chain-enhancer of activated B cells (NF-κB)-associated signaling and mitochondrial death pathway [43,44,45] (Table 1). In addition to NS4B, HCV NS2 protein has been proven to reduce protein synthesis by induction of ER stress and UPR through increasing eIF2a phosphorylation and by transactivations of GRP78, ATF6, and the CCAAT/enhancer binding protein (c/EBP) homologous protein (CHOP), a downstream transcriptional factor of UPR [46] (Table 1). Apart from the NS proteins in the activation of ER stress and UPR, HCV structural proteins, including E1 and E2 proteins, are also able to modulate UPR by regulating PERK and its associated downstream effectors and activating the transcription of CHOP [47,48] (Table 1). Collectively, these studies suggest that HCV induces ER stress and activates UPR signaling to benefit viral replication and modulate the protein synthesis and folding in the infected cells.

However, whether ER stress and UPR are activated in the cell context under which the complete viral life cycle is encountered and how UPR signaling functions in HCV growth remain largely unknown until the HCV genotype 2a JFH1-based cell culture (HCVcc) model was established by T. Wakita [49]. Taking the advantage of the HCVcc infectious model, Sir *et al*. first showed that transfection of JFH1 RNA into Huh7 cells can simultaneously activates the three modules of UPR signaling, and knockdown of genes involved in each arm of UPR strikingly inhibited the viral RNA level [50] (Table 1). Their results suggest that the overt activation of UPR in cells expressing the full-length viral genome is indispensable for HCV RNA replication. Along with this study, we further demonstrated that infection of HCVcc into Huh7 cells leads to activation of the three UPR modulators to transactivate the CHOP gene expression, which in turn triggers autophagic response to enhance viral RNA replication [51] (Table 1). These two studies provide the first line of evidence that the three UPR signaling modules are activated in HCV-expressing cells. Very recently, the ERAD pathway downstream of UPR has been demonstrated to be induced by HCV JFH1 infection and has been implied to participate in the quality control of E2 glycoprotein and virus particle production [52] (Table 1). Moreover, Joyce *et al*. used the severe combined immunodeficiency disorder (SCID) mice transgenic for urokinase plasminogen activator (uPA) under control of the albumin (Alb) promoter that transplanted with human hepatocyte model (SCID/Alb-uPA chimeric mice model) for HCV infection to test whether HCV can activates ER stress *in vivo* [53]. By inoculating the transgenic mice with the HCV genotype 1a patient’s serum, the ER chaperon GRP78 and an apoptotic protein, Bcl-2-associated protein X (BAX) were upregulated whereas expressions of NF-κB and B-cell lymphoma-extra large protein (BCL-xL), two anti-apoptotic molecules, were decreased, indicating that HCV-induced ER stress sensitizes the infected cells to apoptosis in the *in vivo *animal model [53] (Table 1). Merquiol *et al*. also showed a similar result that HCV induces upregulation of UPR downstream target genes in the HCV-transgenic mice in which the entire HCV genotype 1b viral genome is expressed under alpha-1 antitrypsin promoter [54] (Table 1). Importantly, the ER stress-UPR signaling has been shown to be induced in the liver biopsy of patients with chronic HCV infection, as demonstrated by the activation of the three UPR sensors [55] (Table 1). These studies collectively indicate that HCV indeed activates ER stress and UPR *in vivo*. 

**Table 1:** Summary of HCV-regulated ER stress and UPR.

Approach/Model	Characteristics	Functional impacts	Reference
Overexpression of HCV NS4B/ Yeast-two hybrid; Coimmuno-precipitation in human cervical cancer cell, HeLa	Physical interaction between HCV NS4B and ATF6β Colocalization of HCV NS4B with ATF6β	Modulation of ATF6-mediated UPR	Tong *et al*. [41]
HCV-Con1 (1b) replicon transfection/ Human hepatoma cell Huh7	Induction of ATF6 cleavage Increased transcriptional level of Grp78 Downregulation of eIF2α phosphorylation	Activation of cap-independent and cap-dependent translation	Tardif *et al*. [42]
Overexpression of HCV E2/ Human embryonic kidney (HEK) 293 and HeLa cell lines	Binding of E2 to PERK Inhibition of PERK activity by E2 Downregulation of eIF2α phosphorylation by E2	Establishment of persistent infection by E2-mediated counteraction against ER stress	Pavio *et al*. [48]
Overexpression of HCV NS4B/Huh7 and HeLa	Induction of ATF6 cleavage Alternative spicing of XBP1 mRNA Activations of transcriptional levels of ATF6, Grp78, and caspase 12	Benefit to viral RNA replication	Zheng et al. [44]
Overexpression of HCV E1 and E2/ HeLa and Mouse embryonic fibroblast (MEF)	Activations of the CHOP and Grp78 mRNA levels by HCV E1 and E2 Induction of CHOP and Grp78 protein level by HCV E1 and E2 Enhancement of alternative splicing of XBP1 mRNA by HCV E1 and E2	Activation of UPR and ERAD by HCV	Chan and Egan [47]
HCV-JFH1 (2a) viral RNA transfection/ Human hepatoma cell Huh7.5-1	Induction of PERK phosphorylation by HCV viral RNA transfection Activation of eIF2α phosphorylation by transfection of HCV viral RNA Upregulation of CHOP, ATF4, and Grp78 expressions	Promotion of viral RNA replication; Activation of autophagy	Sir *et al*. [50]
Overexpression of HCV NS4B/ Human hepatic cell lines Hep3B, HepG2, and Huh7	Induction of ATF6 cleavage by HCV NS4B Induction of alternative splicing of XBP1 mRNA by HCV NS4B Stimulation of ROS and perturbing calcium homeostasis	Modulation of intracellular NF-κB signaling	Li *et al*. [43]
HCV-H77c (1a) infection/ Chimeric SCID/Alb-uPA mice transplanted with human hepatocytes	Increased level of Grp78 Enhanced level of apoptotic protein, BAX Decreased NF-κB and BCL-xL levels	Sensitization of the infected cells to apoptosis	Joyce *et al*. [53]
Overexpression of HCV NS2; full-length and subgenomic HCV (1b) replicons transfection/ Huh7 and Huh7.5	Induction of eIF2α phosphorylation by HCV NS2 Upregulation of CHOP and Grp78 mRNA levels by HCV NS2 Induction of ATF6, Grp78, and CHOP by transfection of HCV full-length and subgenomic replicons	Modulation of IRES-mediated translation	Von derm Bussche *et al*. [46]
Liver biopsy specimens from patients with chronic HCV infection	Activation of the three ER stress sensors ATF-6, IRE1, and PERK by chronic HCV infection Induction of Grp78 and ATF4 by chronic HCV infection	Modulations of inflammation and apoptosis	Asselah *et al.* [55]
HCV-JFH1 (2a) infection/Huh7	Activation of the three ER stress sensors ATF-6, IRE1, and PERK by HCV infection Induction of CHOP by HCV infection	Promotion of viral RNA replication; Activation of autophagy; Suppression of antiviral innate immunity	Ke and Chen [51]
HCV-JFH1 (2a) infection/Huh7; HCV-transgenic mice	Activation of the 3 arms of the UPR by HCV infection Upregulation of UPR downstream genes by HCV infection Chronic ER stress and activation of UPR downstream genes in HCV-transgenic mice	Counteracting cellular ER stress and adaptation of UPR	Merquiol *et al*. [54]
HCV-JFH1 (2a) infection/ Huh7 and Huh7.5-1	Induction of alternative splicing of XBP-1 mRNA by HCV infection Activation of ERAD by HCV infection Promotion of ERAD signaling by enhancing expressions of ERAD downstream molecules.	Increment of HCV envelope glycoproteins degrdation	Saeed *et al.* [52]

### 2.2. Autophagy

**Figure 4:**
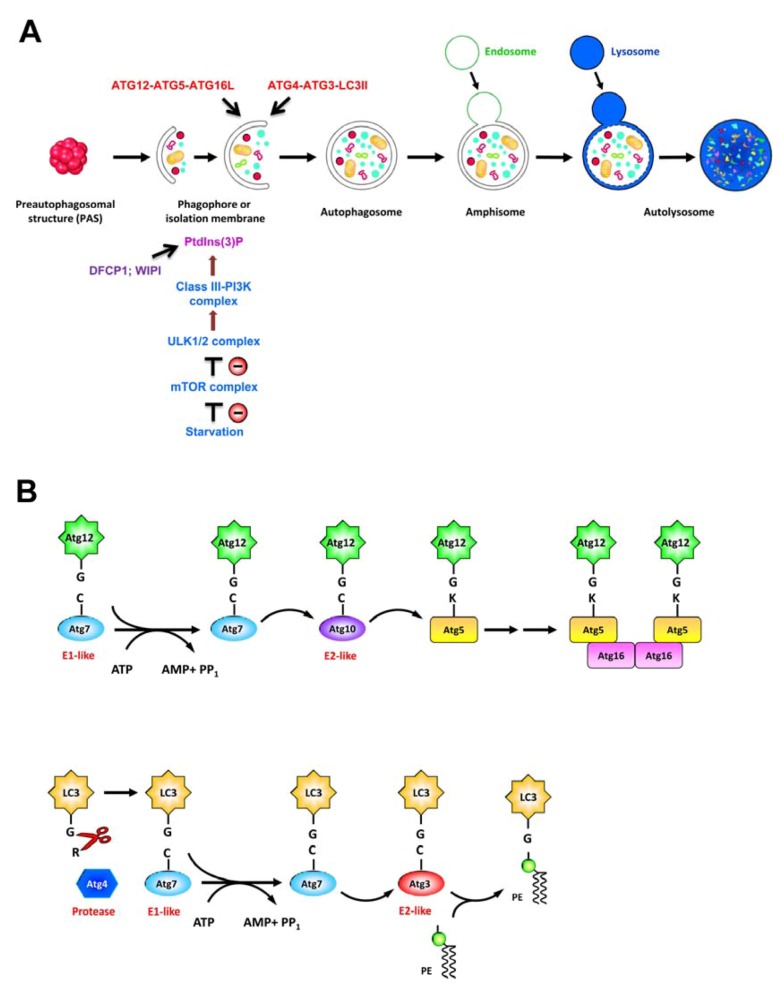
Schematic diagram of autophagy process. (A) Current model of the cellular events involved in autophagic pathway, include membrane nucleation, elongation and completion of autophagosome, and maturation of autolysosome. In response to nutrient starvation, the mammalian target of rapamycin (mTOR) complex dissociates from the unc-51 like-kinase 1 or 2 (ULK1 or ULK2) complex, leading to dephosphorylation, activation, and translocation of ULK1/2 complex to ER. Then the ER-localized ULK1/2 complex activates the class III phosphatidylinositol-3-OH kinase (class III-PI3K) complex, leading to generation of PtdIn(3)P which recruits double-FYVE-containing protein 1 (DFCP1) and WD-repeat domain PtdIns(3)P-interacting (WIPI) proteins onto the isolation membrane of ER, also called omegasome. Two ubiquitin-like conjugation systems including ATG12-ATG5-ATG16L and ATG4-ATG3-LC3II pathways are coordinated with the elongation and enclosure of autophagosome. Finally, the autophagosome fuses with lysosome, and the sequestrated materials are degraded in the autolysosome. (B) Ubiquitin-like conjugation cascades of ATG12-ATG5-ATG16L and ATG4-ATG3-LC3II. Upper panel: ATG12 is first activated by ATG7 (E1-like enzyme) through the formation of thioester bonding between the C-terminal glycine of ATG12 and the active site cysteine residue of ATG7 in an ATP hydrolysis-dependent manner. Then the activated ATG12 is transferred to ATG10 (E2-like enzyme) via a thioesterification reaction, and finally forms a conjugate with ATG5. The ATG5-ATG12 conjugate interacts with ATG16 to form an ATG5-ATG12-ATG16 complex. Bottom panel: LC3 is processed by the cysteine protease ATG4, resulting in exposure of the C-terminal glycine of LC3. The exposed glycine of LC3 is activated by ATG7 (E1-like enzyme), transferred to ATG3 (E2-like enzyme), and then covalently linked to phosphatidylethanolamine (PE) to form the lipidated form of LC3, *i.e., *LC3-II. The ATG12-ATG5-ATG16L is present on the outer membrane, and LC3II is present on both the inner and outer membranes of autophagosome.

Autophagy is an intracellular “self-eating” process that targets the cytoplasmic components via the double membrane vacuoles to lysosome for degradation. As a regulated catabolic process, autophagy exerts a broad range of impacts on cellular pathways to counteract stresses, e.g., nutrient starvation, accumulation of damaged cytoplasmic components, pathogen infection, or ER stress, thus maintaining cellular homeostasis and enabling cell survival [56,57]. Activation of autophagy initiates with the sequestration of cytoplasmic components within a membranous structure, called isolation membrane (IM) or phagophore, that expands to form a double-membraned autophagosome (Figure 4A). Finally, autophagosome then fuses with endosome and lysosome, forming the autolysosome where the engulfed material is degraded by lysosomal proteases [56,57] (Figure 4A). 

Induction and completion of autophagic process rely on stringent regulation of signal transduction pathways and more than 30 autophagy-related genes (ATGs) whose products function in this pathway [57,58]. Under nutrient deprivation, autophagy is initially activated by repressing the mammalian target of rapamycin (mTOR), a serine/threonine protein kinase involved in regulation of cell growth and metabolism. The autophagy pathway involves a series of vacuole regeneration processes including nucleation and elongation of IM/phagophore, formation of autophagosome, and maturation of late-stage autolysosome (Figure 4A). At least four complexes of ATG proteins have been identified to regulate the autophagic process; these modules include the unc-51 like-kinase 1 or 2 (ULK1 or ULK2) complex (ULK1-ATG13-FIP200-ATG101), the class III phosphatidylinositol-3-OH kinase (class III-PI3K) complex (PI3K-Vps15-Beclin-ATG14), the ubiquitin-like protein (UBL) conjugation cascade consisting of the ATG12-ATG5-ATG16L and ATG4-ATG3-LC3II complexes, and the lysosome-associated membrane protein family (LAMP1, LAMP2, and Rab7) [57,58]. At the stage of nucleation of IM/phagophore, inhibition of the mTOR by nutrient starvation results in translocation of ULK 1/2 complex from cytoplasm to a unique ER membrane-associated compartment (Figure 4A). The translocation of ULK1/2 complex onto ER-derived membrane leads to recruitment of PI3K complex, producing phosphatidylinositol-3-phosphate[PtdIns(3)P] [59]. Then the newly-generated PtdIns(3)P recruits the downstream effectors such as the double-FYVE-containing protein 1 (DFCP1) and WD-repeat domain PtdIns(3)P-interacting (WIPI) family proteins (Figure 4A), resulting in formation of an ER-associated Ω-like structure called omegasome [60]. When nucleation is completed, the membrane of IM/phagophore elongates and encloses to form an autophagosome, and this process requires ATG12-ATG5-ATG16L and ATG4-ATG3-LC3II two UBL complexes (Figure 4A). Firstly, ATG12 is conjugated to ATG5 through the concerted action of ATG7 (E1-like) and ATG10 (E2-like) two enzymes (Figure 4B, upper panel). Subsequently, the ATG5-ATG12 conjugate associates with ATG16L to form a trimeric complex. Secondly, the microtubule-associated protein 1 light chain 3 (LC3), *i.e.*, ATG8, is cleaved at its C-terminus through the proteolytic activity of ATG4, and the cleaved LC3 is then conjugated to phosphatidylethanolamine (PE), a major constituent of cellular membrane, to form the lipidated form of LC3, *i.e*., LC3-II, through the enzymatic activities of ATG7 (E1-like) and ATG3 (E2-like) [61] (Figure 4B, bottom panel). At the final stage of autophagy, the mature autophagosome fuses with vacuoles derived from endocytosis and lysosome to form autolysosome, in which the sequestered materials are degraded and recycled for further use by cells [57] (Figure 4A). Apart from its role in the maintenance of nutrient homeostasis, autophagic process has also been identified in various biological processes such as development, differentiation, and tissue regeneration [62]. In addition, autophagy also plays important functions in the pathogenesis of various diseases, including cancer and microbial infection [56,57,63]. 

Viruses such as poliovirus, rhinovirus, and mouse hepatitis virus have been shown to induce autophagy to benefit their life cycle; these viruses subvert the autophagic machinery by exploiting the autophagosomal membrane as their replication site [64,65,66]. In contrast to its pro-viral role, autophagy could exert its anti-viral activity against infection of other viruses, e.g., tobacco mosaic virus and Sindbis virus, by engulfing and destroying infecting virus through xenophagy and apoptotic death of infected host cells [67,68,69]. Thus, autophagy can function as a means to restrict viral replication and protect host cells against viral pathogenesis [70]. Also, host cells can usurps autophagy machinery to enhance Toll-like receptors (TLRs)-mediated innate immune response and facilitate the presentation of antigens derived from viruses like vesicular stomatitis virus (VSV) and Epstein-Barr virus (EBV) onto MHC class II molecule [71,72].

Several members of Flaviviridae family including HCV, Dengue virus (DENV), and Japanese encephalitis virus (JEV) have been reported to induce autophagic response [50,51,73,74,75,76,77,78]. Also, emerging lines of evidence have indicated that autophagy is activated by HCV [50,51,73,74] (Table 2). Ait-Goughoulte and colleagues first showed that serial passage of immortalized human hepatocytes (IHH) transfected by the HCV genotype 1a (clone H77) genome induces accumulation of autophagic vacuoles and formation of GFP-LC3-labeled punctate dots, two major characteristics of autophagic process [73] (Table 2). This HCV-activated autophagic response in IHH was in accordance with upregulations of the ATG5-ATG12 conjugate and Beclin [73]. The HCV-induced autophagosome formation was also detected in the Huh7 cells transfected with HCV genotype 2a JFH1 viral RNA, as demonstrated by the expression of LC3-II and the GFP-LC3-labeled punctate structure [50]. Because of the lack of enhanced autophagic degradation of a long-lived protein p62 and the inefficient maturation of autophagic vacuoles in cells expression HCV JFH1 viral RNA, Sir *et al*. proposed that HCV-induced autophagic response undergoes an incomplete process which is hampered at the maturation stage of autolysosome [50] (Table 2). Moreover, the authors demonstrated that suppression of ATG7 or LC3 dramatically inhibited the replication of HCV viral RNA [50], implying that autophagy may participate in the replication of viral RNA. Very recently, the authors further demonstrated that the membranous structure of viral-induced autophagosome may be utilized as the replication site of HCV JFH1 replicon viral RNA [79]. In JFH1 virus-infected cells, Dreux *et al.* further demonstrated that HCV enhances autophagy to regulate the translation of incoming viral RNA [74] (Table 2). On the other hand, we first showed that HCV JFH1 infection activates autophagy to complete autolysosome formation, as demonstrated by (1) the presence of both the initial- and late-stage autophagic vacuoles (AVi and AVd, respectively); (2) accumulation of HCV-induced LC3-II by interfering with autolysosome maturation; and (3) colocalization of HCV-induced autophagic vacuoles with lysosome [51,74]. By gene silencing and pharmacological inhibitor experiments, we further demonstrated that HCV-triggered autophagic response represses the innate antiviral immunity to promote viral RNA replication [51,74] (Table 2). Interference with autophagy significantly enhanced HCV pathogen-associated molecular pattern (PAMP, 3’-UTR and poly-U/UC regions of HCV genome)-triggered IFN-β activation and ISG production [51]. Reciprocally, activated autophagic response by chemical inducers (e.g., rapamycin and Tunicamycin) or nutrient starvation dramatically inhibited the HCV-PAMP-mediated innate immune response and this suppression can be orchestrated by treatment with an autolysosome maturation inhibitor, chloroquine (CQ) [51]. In line with our study, Shrivastava and colleagues also found that knockdown of Beclin or ATG7 not only inhibits HCV growth but also potentiates the activation of expressions of IFN and ISGs in the HCV-infected human immortalized hepatocytes [80] (Table 2). Therefore, these results collectively indicate that HCV exploits autophagy to escape innate antiviral immunity via an unidentified mechanism, thus promoting viral infection.

Apart from its role in viral RNA replication, several papers have reported additional roles of autophagy in the assembly of HCV infectious virus and surveillance of the HCV-infected host cells [81,82] (Table 2). Tanida and colleagues showed that silencing of ATG7 and Beclin moderately decreased the level of extracellular infectious HCV particle, but did not affect the intracellular amounts of viral RNA and proteins [82] (Table 2). Their observation implies that HCV-induced autophagic machinery may regulate the release process of HCV particle. On the other hand, Taguwa *et al*. demonstrated that interference with the HCV Con1 (genotype 1b)-induced autophagy by ectopic expression of a protease-inactive mutant ATG4B^C47A^ leads to severe cytoplasmic vacuolation and cell death [81] (Table 2), suggesting that HCV may utilize the autophagic process to circumvent the adverse cellular response against stresses induced by viral replication and thus promote survival of infected cells. 

The molecular mechanism underlying how HCV activates autophagy has been poorly understood so far. Several studies have shown that ER stress and UPR could activate autophagy [83,84,85]. In the case of HCV, two independent studies demonstrated that HCV activates UPR to induce the autophagy [50,51]. However, the conflicting results reported by Mohl *et al.* indicated that the HCV-induced autophagic response precedes the stimulation of UPR, suggesting that HCV activates autophagy in an UPR-independent manner [86]. On the other hand, Su *et al*. showed that HCV NS4B can induce autophagy signaling through the interaction with Rab5 and Vps34 [87] (Table 2). On the other hand, HCV infection has been shown to activate autophagy through transcriptionally upregulating Beclin level and activating mTOR signaling [88] (Table 2). In this study, the authors showed that HCV NS5A is sufficient to trigger autophagic response [88] (Table 2). Very recently, Gregoire and colleagues showed that several RNA viruses, including HCV, may target autophagy-associated proteins via the immunity-associated GTPase family M protein (IRGM) [89]. Knockdown of IRGM strikingly impaired the HCV-induced autophagic response as well as the production of infectious viral particle, indicating that IRGM may play a central role in the regulation of HCV-triggered autophagy [89]. Nonetheless, additional studies will be necessary to determine how HCV RNA or proteins exploit the cellular signaling pathway to activate autophagy. 

**Table 2:** Summary of HCV-activated autophagy.

Approach/Model	Characteristics	Functional impacts	Reference
HCV-H77 (1a) viral RNA transfection/ Immortalized human hepatocytes (IHH)	Formation of GFP-LC3 punctate structure TEM analysis of autophagic vacuoles. Upregulated levels of Beclin and ATG5-ATG12 conjugate	Viral RNA replication	Ait-Goughoulte *et al*. [73]
HCV-JFH1 (2a) viral RNA transfection/ Huh7.5	Upregulation of LC3B-II No colocalization of accumulated autophagosome with lysosome UPR-mediated autophagic activation Lack of enhanced autophagic degradation Incomplete autophagic process	Viral RNA replication	Sir *et al*. [50]
HCV-JFH1 (2a) infection/ Huh7	Increased lipidation of LC3B-II Formation of GFP-LC3 punctate structure Requirement of autophagy for initial replication of HCV, but not for the maintenance of existing replicating genome No colocalization of autophagic vacuoles with viral proteins	Translation of incoming viral RNA	Dreux *et al*. [74]
HCV-JFH1 (2a) infection/ Huh7.5-1	Formation of GFP-LC3 punctate structure No colocalization of autophagic vacuoles with viral proteins	Viral particle assembly	Tanida *et al.* [82]
HCV-JFH1 (2a) infection/ Huh7	Transient association of ATG5 with NS5B and NS4B Colocalization of ATG5 with membranous web	Organization of replication site	Guevin *et al*. [90]
HCV-JFH1 (2a) infection/ Huh7	TEM analysis of early- and late-stage autophagic vacuoles Colocalization of accumulated autophagosome with lysosome Increased accumulation of LC3B-II by CQ or BAF-A1 treatment Complete autophagic process UPR-mediated autophagic activation	Viral RNA replication; Suppression of antiviral innate immunity	Ke and Chen [51]
HCV-H77 (1a); HCV-JFH1 (2a) infection/ IHH	Inhibition of HCV growth by knockdown of Beclin and ATG7 in the HCV-infection IHH Enhanced interferon response in the HCV-infected cells knockdown of Beclin and ATG7 Activated caspase-dependent apoptosis by knockdown of Beclin and ATG7 in the HCV-infection IHH	Viral RNA replication; Suppression of antiviral innate immunity	Shrivastava *et al*. [80]
HCV-JC1 (2a) infection; overexpression of HCV NS4B/Huh7.5	Induction of autophagy by HCV; NS4B Mapping of HCV NS4B amino acids 1-190 for autophagic activation Requirement of Rab5 and PI3K for autophagic activation	Organization of viral replication site	Su *et al*. [87]
HCV-Con1 (1b) and JFH1 (2a) Replicon RNA transfection / Huh7	Induction of autophagy in HCV replicon cells Impaired autophagic flux in Con1 replicon cells, but not in JFH1 replicon cells Enhanced secretion of immature cathepsin B in Con1 replicon cells Requirement of autophagy for cell survival	Protection of host cells from viral infection-induced death	Taguwa *et al*. [81]
HCV-JFH1 (2a) replicon RNA transfection/ Huh7.5	Suppression of viral RNA replication by knockdown of LC3 and ATG7 Colocalization of NS5A, NS5B, and nascent viral RNA with autophagosome Failure of inhibition on autophagy activation by interference with class III-PI3K activity	Replication site of viral RNA	Sir *et al*. [79]
HCV-JFH1 (2a) full-length and subgenomic HCV replicon RNA transfection/ Huh7; HCV-transgenic mice	Increased ROS in mitochondria in the HCV-expressing cells Enhanced autophagic response by expression of HCV NS proteins Alteration of antioxidant response by upregulation of antioxidant enzymes in HCV NS proteins-expressing cells	Regulation of oxidative response.Mitochondria-mediated cytopathic effects	Chu *et al*. [91]
HCV-JFH1 (2a) infection/ IHH	Transactivation of Beclin expression by HCV infection HCV-induced autophagy activation is independent of Bcl2-Beclin dissociation Activation of autophagy by HCV in not through inhibition of mTOR activity	Viral RNA replication	Shrivastava *et al*. [88]
HCV-Con1 (1b) and JFH1 (2a) replicon RNA transfection / Huh7 and Huh7.5-1	An inverse correlation between microvesicular steatosis and the level of autophagy Colocalization of autophagic vacuoles with LDs Impaired autophagy causing cholesterol accumulation	Metabolism of LDs; Regulation of lipid storage	Vescovo *et al*. [92]
HCV-JFH1 (2a) infection/ Huh7.5	Interaction of IRGM with ATG proteins Decreased HCV-induced autophagic response by IRGM knockdown Impaired HCV growth by IRGM silencing	Promoting viral particle production; Regulation of antiviral response	Gregoire *et al.* [89]
HCV-JFH1 (2a) infection/ Huh7; Huh7.5	Occurrence of HCV-induced autophagy earlier than UPR stimulation Activation of autophagy by HCV subgenomic replicon. Independence of HCV-induced autophagyof UPR	No apparent role of HCV-induced autophagosomal membrane in HCV replication	Mohl *et al*. [86]
HCV-JFH1 (2a) viral RNA transfection/ Huh7	Disturbance of glucose homeostasis by HCV. Dysregulation of insulin signaling by HCV. Inhibition of HCV-induced autophagy by 3-methyladenine. Interaction of Beclin with phosphorylated IRS-1 (Ser312)	Dysregulation of glucose homeostasis; Induction of insulin resistance	Das *et al*. [93]

### 2.3. Apoptosis

Triggering virus-infected cell to death via apoptotic process is an important strategy of host to protect itself against viral infection [94,95]. Apoptosis is induced through two major pathways, including the mitochondria-mediated intrinsic pathway and the tumor necrosis factor (TNF) family-induced extrinsic pathway [96,97,98,99]. Mitochondrial apoptosis is initially triggered by various stress signals and that mitochondria integrate cell death signals through the imbalance of proapoptotic regulators, the B-cell lymphoma-2 (Bcl-2) family members, such as Bcl-2, BAX, and the BH3-interacting death agonist protein (BID) [99,100]. The accumulation of proapoptotic proteins on mitochondria causes the outer mitochondrial membrane to be permeable [99,100]. The increase in mitochondrial permeability triggers the release of cytochrome C and ultimately results in caspase activation [99,100]. The extrinsic cell death pathway is triggered by the TNF superfamily of cytokines, which activate signaling pathways for cell survival, apoptosis, inflammatory responses, and cellular differentiation [98]. Most of the TNF family proteins are potent inducers of signaling that triggers activation of NF-κB-mediated cell survival signaling whereas some of the members of TNF superfamily can induce apoptosis by binding to the so-called death receptors, e.g., TNF receptor 1 (TNFR1), Fas/CD95, TNF receptor apoptosis-inducing ligand (TRAIL) receptor 1 (TRAILR1/DR4), or TRRAILR2/DR5 [101,102]. The binding specificities of ligands to these receptors are determined by the typical amino-terminal cysteine-rich domains (CRDs) [102]. Engagement of ligand to receptor delivers an immediate proapoptotic signal through the binding of a death domain (DD) within receptors to the adaptor protein FADD [98,103]. The FADD in turn activates caspase 8 via its death effector domain (DED) and then the activated caspase-8 subsequently triggers the activation of downstream caspases that participate in the execution of the apoptotic process [98,103].

Several studies have shown that HCV viral proteins may modulate cell apoptosis. Some viral proteins, such as core, E1, E2, NS3, NS4A, NS4B, NS5A, and NS5B have been demonstrated to trigger apoptosis through increasing mitochondrial permeability or inducing TNF-downstream caspase 8 activation [45,104,105,106,107,108,109,110] (Table 3). On the contrary, the antiapoptotic functions of HCV viral proteins, including core, E2, NS2, NS3, and NS5A, have also been reported [111,112,113,114] (Table 3). Due to these controversies, whether HCV induces or antagonizes apoptotic cell death needs to be studied in cells harboring the complete viral life cycle rather than by overexpression of individual viral proteins. Nomura-Takigawa and colleagues first showed that replication of HCV replicon RNA triggered apoptotic cell death through NS4A-mediated mitochondrial damage and release of cytochrome C [108] (Table 3). Further study by Deng *et al*. demonstrated that infection of the HCV J6/JFH1 chimeric strain into Huh7.5-1 cells induces apoptosis which is associated with activation of caspase 3 and the cleavage of poly(ADP-ribose) polymerase (PARP), a downstream substrate of the activated caspase 3 [115] (Table 3). Moreover, the authors pointed out that HCV-induced apoptosis is mediated by the disruption of mitochondrial transmembrane potential and the causative oxidative stress [115]. On the other hand, two studies demonstrated that HCV infection can induce the TNF-mediated cell apoptosis through upregulation of TRAIL and its receptors, DR4 and DR5 [116,117] (Table 3). These studies collectively suggest that HCV infection could sensitize host cells to apoptotic death though altering the mitochondrial dynamics and enhancing the expression of TNF-associated death receptors. 

**Table 3:** Summary of HCV and modulation of apoptosis.

Approach/Model	Characteristics	Functional impacts	Reference
Overexpression of HCV core/ Human breast cancer cell line MCF7	Inhibition of TNF-α-induced cytotoxicity by HCV core Inhibition of TNF-α-induced DNA fragmentation and the cleavage of PARP by HCV core	Inhibition of TNF-α-mediated apoptosis	Ray *et al*. [111]
Overexpression of HCV core/ HeLa and HepG2 cell lines	Interaction of the HCV core protein with the cytoplasmic domain of TNFR1 Enhanced TNF-α-induced apoptosis by HCV core	Activation of TNF-α-induced apoptotic signaling	Zhu *et al*. [110]
Overexpression of HCV NS5A/ Monkey kidney cell line COS7 and Hep3B	Binding of HCV NS5A to p53 Suppression of p21/waf1 expression by HCV NS5A Inhibition of p53 -mediated transcriptional transactivation and apoptosis	Inhibition of p53 downstream apoptotic signaling	Lan *et al*. [114]
Transfection of HCV viral replicon RN/ Huh7	Inhibition of cleavage of procaspase-3 and procaspase-9 by HCV E2 Inhibition of cleavage of PARP by HCV E2 Interference with TRAIL-mediated apoptosis by HCV E2	Inhibition of TNF-α-mediated extrinsic apoptosis	Lee *et al*. [112]
Overexpression of HCV core/ Huh7 and HepG2; HCV core-transgenic mice	Induction of the proapoptotic factor CHOP, translocation of BAX to mitochondria, depolarization of mitochondrial membrane, release of cytochrome c, caspase-3 and PARP cleavage by HCV core Induction of ER stress and apoptosis in HCV core transgenic mice	Activation of apoptotic cell death	Benali-Furet *et al*. [104]
Overexpression of HCV NS3/ Huh7, HepG2, and HEK293	Activation of caspases by HCV NS3 Interaction of HCV NS3 with caspase 8 Colocalization of HCV NS3 with caspase 8 Sensitization of cells to Fas-induced apoptosis by HCV NS3	Promotion of caspase 8-mediated apoptosis	Prikhod'ko *et al*. [109]
Baculovirus-mediated expression of HCV E1/ Insect Sf9 cell	Induction of DNA fragmentation by HCV E1 Induction of cytotoxicity by HCV E1	Activation of apoptosis	Ciccaglione *et al*. [106]
Overexpression of HCV E2/ Huh7	Inhibition of cell proliferation by HCV E2 Induction of DNA fragmentation by HCV E2 Activation of caspases by HCV E2	Sensitization of the cells to apoptosis	Chiou *et al*. [105]
Overexpression of HCV NS4A; subgenomic HCV (1b) replicons transfection/ Huh7	Alteration of mitochondrial distribution by HCV NS4A Induction of mitochondria damage by HCV NS4A Activation of caspase 3 by HCV NS4A Induction of mitochondria-mediated apoptosis by expression of HCV replicon	Promotion of mitochondria-mediated intrinsic apoptotic cell death	Nomura-Takigaw et al. [108]
Overexpression of HCV core/ HEK293T	Translocation of Bax from cytosol to mitochondria by HCV core Disruption of mitochondrial permeability by HCV core Enhanced release of cytochrome C by HCV core Activation of caspases 9 and 3 by HCV core	Promotion of mitochondria-mediated intrinsic apoptotic cell death	Lee et al. [107]
Overexpression of HCV NS3/ Huh7 and HeLa cells	Interaction of HCV NS3 with p53 Impaired complex formation of HCV NS3 with p53 by single-point mutations of NS3 at position 106 from Leu to Ala (L106A) Interference with anti-apoptotic activity by L106A mutation on NS3	Inhibition of apoptosis by HCV NS3 required serine protease activity	Tanaka et al. [113]
HCV- chimeric J6/JFH1 (2a) infection/ Huh7.5-1	Activation and nuclear translocation activated caspase 3 by HCV infection Enhanced the cleavage of PAPR by HCV infection Accumulation of BAX on mitochondria by HCV infection	Activation of mitochondria-mediated intrinsic apoptosis by HCV infection	Deng et al. [115]
HCV-JFH1 (2a) infection/ Huh7 and LH86 cell lines	Induction of DNA fragmentation by HCV infection Activation of TRAIL-mediated apoptosis by HCV infection Induction of DR4 and DR5 mRNA levels by HCV infection	Activation of death receptor-mediated extrinsic apoptosis by HCV infection	Zhu et al. [117]
HCV-JFH1 (2a) infection; replicon viral RNA transfection/ Huh7	Induction of TRAIL-mediated apoptosis by HCV replication Upregulation of DR4 and DR5 mRNA and protein levels by HCV infection HCV-induced DR4 and DR5 upregulation is dependent on MEK1 activation	Sensitization of the virus-infected cells to TRAIL-mediated extrinsic apoptotic pathway	Deng et al. [116]

### 2.4. Cell Cycle Arrest and DNA Damage Mitogenic Signaling, and PI3K Pathway

Progression of cell cycle is tightly controlled by cyclin-dependent kinase (CDK) complexes whose activities are positively or negatively regulated by cyclins and CDK inhibitors (CKIs), respectively [118]. CKIs, such as p21 and p16, play an important role in cell cycle progression by coordinating internal and external signals and impeding proliferation at several restriction points, termed as checkpoints [118]. The checkpoint in each stage ensures the proper progression of cell cycle [118]. Virus infection often leads to cell cycle arrest through altering the activity of cell cycle regulators [119,120]. For example, the viral infectivity factor (Vif) encoded by human immunodeficiency virus type 1 (HIV-1) has been shown to induce G2 arrest via its interaction with p53/murine double minute 2 (MDM2) axis to promote HIV replication [121]. 

HCV core protein is shown to repress the expressions of tumor suppressor p53 and CKI p21, leading to CDK2 activation and phosphorylation of the retinoblastoma gene product, RB, in liver and non-liver cells [122] (Table 4). The HCV core-induced phosphorylation of RB enhances the DNA binding ability of E2F transcriptional factor 1 (E2F-1) and activates the gene expression of S phase kinase-interacting protein 2, suggesting that HCV core protein may be involved in cell proliferation [122]. On the other hand, HCV NS5B has been demonstrated to interact with RB and targets it to proteolysis by an E6-associated protein (E6AP) E3 ligase-dependent pathway in Huh7 cells harboring HCV replicon RNA [123,124] (Table 4). HCV NS5B-induced RB degradation not only activates E2F-responsive promoter, but also stimulates S phase entry and cell proliferation [123,124]. Moreover, the authors further demonstrated that the binding capacity of NS5B to RB may regulate host gene expressions to promote viral replication through modulating the cytosolic RB abundance [125] (Table 4). Apart from the alteration of S phase by HCV, it has been reported that HCV infection leads to chromosomal polyploidy and impairs mitotic checkpoint by reducing RB phosphorylation and enhancing E2F-1 and mitotic arrest deficient 2 (Mad2) expressions in human peripheral blood mononuclear cells (PBMCs) [126] (Table 4). Moreover, these negative effects on mitotic progression can be mimicked by overexpression of HCV core protein alone [126], suggesting that core protein exerts its inhibitory role in the mitotic checkpoint of HCV-infected cells. On the other hand, Kannan and colleagues showed that HCV H77/JFH1 chimeric virus infection in Huh7 cells reduces cell proliferation which is correlated to the viral antigen abundance [127] (Table 4). A decrease in cell proliferation of the HCV-infected cells is associated with the reduction in cell population of G1/S phase and accumulation of G2/M phase cells, suggesting that HCV infection interferes with the initiation of mitosis [127]. These results conclude that HCV infection can differentially induce the arresting effect at different stages of cell cycle. 

Despite the role of modulating cell cycle progression, HCV was shown to be involved in DNA damage response. The HCV NS3/4A has been demonstrated to interact with ataxia-telangiectsia mutated (ATM), a sensor protein essential for cellular response to DNA damage [128] (Table 4). Overexpression of HCV NS3/4A causes cytoplasmic retention and dephosphorylation of ATM, leading to a defect in DNA repair and sensitization of Huh7 cells to ionization [128]. In addition to the interaction between HCV NS3/4A with ATM, Ariumi and colleagues further demonstrated that HCV NS5B protein specifically binds to ATM and checkpoint kinase 2 (CHK2), and that knockdown of each of these DNA damage sensor and transducer inhibits HCV replication [129] (Table 4). These observations suggest that HCV may exploit the ATM DNA damage response to alter host cellular signaling in favor of HCV growth. In line with this study, Machida *et al*. demonstrated that HCV infection interferes with multiple DNA repair pathways through the interaction of core with Nijmegen breakage syndrome protein 1 (NBS1), a major component of the Mre11/Rad50/NBS1 complex which is responsible for ATM-associated DNA damage response [130] (Table 4). Taken together, these studies reveal the inhibitory role of HCV in the host DNA damage/repair response.

Viruses often subvert host cell mitogenic signaling to promote the surveillance of the infected cells and to establish chronic infection. Mitogen activated protein kinases (MAPKs), members of the serine/threonine kinase family, constitute the mitogenic signaling pathway that regulates cell growth in response to extracellular stimuli. Three arms of mitogenic signaling including *c*-Jun N-terminal kinase (JNK), the extracellular signal-regulated kinase (ERK), and p38MAPK pathways have been identified [131]. HCV has been demonstrated to interact with signal transduction pathway involved in cell survival signaling, including PI3K and MAP kinase pathways. The HCV NS5A protein was first shown to interrupt growth factor-bound protein 2 (Grb2)-mediated ERK signaling pathway via binding to Grb2 adaptor protein [132]. Likewise, two independent groups also demonstrated that ectopic expression of NS5A in cells disrupts the Ras-ERK signaling pathway [133,134]. In the same fashion, HCV NS5A was also shown to inhibit p38MAPK signaling [135,136]. Disturbance of p38MAPK pathway by HCV NS5A leads to decrease in phosphorylation of eIF4E, resulting in stimulation of HCV IRES-dependent and cap-independent protein translation [135,136]. On the contrary to the inhibitory role in these MAPK pathways mentioned above, NS5A exerts its function to activate JNK signaling by interacting with TNF receptor-associated factor 2 (TRAF2) [137,138]. Apart from inhibiting MAPK pathway, HCV has been demonstrated to activate PI3K signaling pathway. HCV NS5A protein was shown to interact with the p85 subunit of PI3K complex to activate the PI3K downstream AKT and BAD signaling molecules [139,140]. Furthermore, the stable complex consisting of HCV NS5A and p85 was also demonstrated in the cells harboring a subgenomic HCV replicon, indicating that replication of HCV viral genome may regulate kinase activity and signaling of PI3K [140]. These studies collectively indicate that HCV modulates MAPK and PI3K pathways to control cell growth signaling of the infected cells, contributing to the pathogenesis caused by HCV.

**Table 4:** Summary of HCV-altered cell cycle progression, DNA damage response, mitogenic signaling, and PI3K pathway.

Approach/Model	Characteristics	Functional impacts	Reference
Retrovirus infection-mediated expression of HCV core/ HepG2 and HeLa cell lines	Inhibition of p53 and p21 expressions by HCV core Activation of CDK2 activity by HCV core Enhanced RB phosphorylation by HCV core Activation of E2F-1through enhancing DNA binding ability by HCV core Induction of S phase kinase interacting protein 2 by HCV core	Modulation of RB/E2F-1-mediated cell cycle progression	Hassan *et al*. [122]
Transfection of HCV viral replicon RNA / Huh7	Inhibition of RB expression by HCV replication Interference with RB expression by HCV NS5B Stimulation of cell proliferation and S phase entry by HCV NS5B	Promotion of G1/S transition	Munakata *et al*. [123]
HCV H77S (1a) and JFH1 (2a) infection/ Huh7 and Huh7.5-1	Enhanced ubiquitination and degradation of RB by HCV NS5B Critical role of the ubiquitin ligase activity of E6AP for the NS5B-dependent ubiquitination of RB	Interference with RB-mediated cell cycle progression	Munakata *et al*. [124]
HCV H77S (1a) and JFH1 (2a) infection/ Huh7 and Huh7.5-1	Reduction of HCV viral RNA replication by mutations in the RB-binding motif of NS5B Failure of downregulating RB expression by HCV viruses with mutations in the LxCxD domain Inhibition of HCV viral RNA replication by knockdown of RB	Modulation of host gene expression by regulating RB abundance	McGivern *et al*. [125]
HCV^+^ PBMC; HCV^-^ PBMC/ B cell-derived HCV infection/ Raji cells HCV JFH1 (2a) infection/ Huh7; HCV core transgenic mice	Inhibition of mitotic checkpoint by HCV infection and HCV core expression Reduced RB transcription and enhanced E2F-1 and Mad2 expression by HCV infection and HCV core expression Increased chromosomal polyploidy by HCV infection	Perturbing mitotic checkpoint; Increasing chromosome instability	Machida *et al*. [126]
HCV^+^ PBMC; HCV^-^ PBMC/ B cell-derived HCV infection/ Raji cells	Enhanced chromosomal aberrations and chromosomal breaks by HCV infection Binding of HCV core to NBS1 Inhibition of the Mre11/NBS1/Rad50 complex formation by HCV core	Inhibiting DNA repair process; Potentiating chromosomal instability	Machida *et al*. [130]
HCV- chimeric H77S/JFH1 (1a/2a) infection/ Huh7.5-1	Inhibition of cell proliferation by HCV infection Decrease in the proportions of cells in G_1_ and S phases with accumulation of cells in G_2_/M phase by HCV infection Activation of caspase 3 by HCV infection	Interfering with G2/M progression	Kannan *et al*. [127]
Overexpression of HCV NS3/4A/ Huh7	Interaction of HCV NS3/4A with ATM Delayed dephosphorylation of the phosphorylated ATM and γ-H2AX following ionizing irradiation by HCV NS3/4A Activation of caspases by HCV E2	Interference with DNA repair process; Sensitization of the cells to DNA damage	Lai *et al*. [128]
Transfection of HCV subgenomic HCV (1b) replicons; HCV JFH1 (2a) infection / Huh7	Suppression of HCV viral RNA replication by knockdown of ATM and Chk2 Interaction of HCV NS3/4A with ATM Interaction of HCV NS5B with ATM and Chk2 Induction of mitochondria-mediated apoptosis by expression of HCV replicon	Promotion of HCV viral RNA replication	Ariumi *et al*. [129]
Vaccinia virus-mediated expression of HCV NS5A/ HeLa	Interaction of NS5A with Grb2 adaptor Inhibition of ERK phosphorylation by NS5A	Interference with ERK signaling; Implication to HCV pathogenesis	Tan *et al*. [132]
Overexpression of HCV NS5A/ HeLa; NIH3T3	Interaction of NS5A with Grb2 Inhibition of ERK phosphorylation by NS5A	Interference with ERK signaling	Georgopoulou *et al*. [133]
Overexpression of HCV NS5A; Transfection of subgenomic HCV replicon / Cos7; 293T; Huh7	Inhibition of MAPK-activated transcriptional factor AP1 by NS5A Interference with ERK signaling by NS5A	Interruption ERK pathway; Inhibition of MAPK-mediated transcription	Macdonald *et al*. [134]
Vaccinia virus-mediated expression of HCV NS5A; Transfection of subgenomic HCV replicon / HeLa S3; Huh7	Inhibition of p38MAPK signaling by NS5A Decrease in eIF4A phosphorylation by expression of NS5A Inhibition of cap-dependent translation by NS5A	Interruption p38MAPK pathway; Inhibition of cap-dependent protein translation	He *et al.* [135,136]
Overexpression of HCV NS5A/ 293T	Interaction of TRAF2 by NS5A Activation of JNK by NS5A	Modulation of TNF signaling; Implication to HCV pathogenesis	Park *et al*. [138]
Tet-Off-mediated expression of HCV NS5A/ HeLa;	Interaction of p85 subunit of PI3K by NS5A Interaction of Grb2 by NS5A Enhanced tyrosine phosphorylation of AKT protein kinase by NS5A Inhibition of BAD by NS5A	Activation of PI3K and AKT signaling; Implication of HCV pathogenesis	He *et al*. [139]
Overexpression of HCV NS5A; Transfection of subgenomic HCV replicon / Cos7; 293T; Huh7	Binding of NS5A to p85 subunit of PI3K Increased AKT phosphorylation by NS5A and HCV replicon RNA	Activation of PI3K and AKT signaling; Implication of HCV pathogenesis	Street *et al*. [140]

## 3. Implication of HCV-Induced Cellular Responses on the Pathogenesis of HCV-associated Liver Diseases

### 3.1. ER stress and UPR

HCV has been well documented to induce ER stress and that activates UPR in the *in vitro* and *in vivo* experimental models [42,50,51,53,54]. Also, the virus-induced UPR is shown to participate in the replication of HCV viral RNA and to promote apoptosis of the infected hepatocytes [50,51,53]. The ER is an important organelle for protein folding, fatty acid synthesis, and cholesterol metabolism. ER stress has been proposed to induce hepatic steatosis by multiple mechanisms. (1) ER stress induces expression of genes that regulate lipogenesis through phosphorylated eIF2a-mediated induction of c/EBP proteins [141]. (2) PERK enhances sterol regulatory element binding protein (SREBP)-SREBP cleavage activating protein (SCAP)-mediated lipid synthesis in ER via attenuating the synthesis of Insig-1 protein [142,143]. (3) PERK-mediated shut down of protein translation along with ERAD impairs lipid secretion through promoting apolipoprotein-B (ApoB) degradation [144,145]. (4) ER stress promotes insulin resistance through JNK-mediated inhibition of insulin receptor substrate-1 (IRS-1) [146,147,148]. On the other hand, accumulating evidence suggests that UPR acts as an important role in the tumorigenesis of liver cancer. For example, the increased expression of a UPR chaperone, Grp78 has been shown to be highly expressed in hepatocellular carcinoma [149,150]. In addition, the elevated Grp78 level positively correlates to the pathological grade in liver cancer [151]. Similarly, activation of ATF6 and XBP-1s has also been demonstrated in human hepatocellular carcinoma [150]. Moreover, overexpression of XBP-1s was shown to promote the neoplastic transformation and development of multiple myeloma [152], suggesting that ER stress-induced accumulation of XBP-1s is an oncogenic factor for tumorigenesis. HCV-induced ER stress is also closely linked to the depletion of calcium, the increase of ROS, and the damage of mitochondria [43,44,45]. The elevation of ROS and the accumulation of damaged mitochondria induce oxidative stress which plays a critical role in a variety of clinical symptom of HCV-associated liver diseases, such as liver injury, chronic inflammation, and malignant transformation [153,154]. In this regard, HCV-induced ER stress and activated UPR are implicated in the pathogenesis of HCV-related liver diseases. Most of this knowledge was accrued from the *in vitro* experimental models overexpressing viral protein and the cell culture model capable of supporting HCV replication cycle. In the future, the dissection of the relationship of virus-induced ER stress and UPR signaling with hepatosteatosis, liver injury, chronic inflammation, and tumorigenesis of hepatocellular carcinoma is urgently needed; this can be performed by examining clinical specimens from HCV-infected patients as well from small animal models which supports the complete HCV life cycle. 

### 3.2. Autophagy

Activation of autophagy by HCV has been demonstrated by several research groups [50,51,73,74]. Also, HCV-induced autophagy was shown to be required for different stages of the viral life cycle, including viral RNA replication, the translation of viral RNA, and the assembly of infectious viral particle [50,51,74,82]. Despite its functional role in viral growth, HCV-activated autophagic response has been suggested to regulate antiviral immune response, modulate apoptotic cell death signaling, promote host cell survival, and counteract virus-induced lipid accumulation [51,81,88,92]. Autophagic response was demonstrated to participate in lipid metabolism through catabolizing lipid droplets (LDs) in liver cells [155]. In addition, autophagy also regulates accumulation of body lipid by controlling adipocyte differentiation [156]. These studies directly imply the possibility that HCV-induced autophagy may contribute to virus-induced alteration of lipid metabolism in host cells. To answer this question, Vescovo and colleagues analyzed the association of activated autophagy with the clinical parameters of lipid metabolism in liver biopsies of HCV chronically infected patients and found that autophagy level is inversely correlated with steatosis in HCV patients [92]. The authors further demonstrated that autophagic process catabolizes lipids in cells harboring HCV replicon RNA replication and impairment of autophagy leads to an elevation of cholesterol level in HCV JFH1-infected cells [92]. This observation implies that HCV-induced autophagy exerts its role to counteract the virus-triggered lipid accumulation. In this regards, it should be noted that deregulation of autophagic response, such as the incomplete autophagy induced by HCV which was reported by Sir and colleague may represent a contrary way to induce excess accumulation of lipid in the infected cells [50].

Despite its catabolic role in lipid metabolic cycle, autophagy was also reported to play an important role in tumorigenesis [157]. Autophagy is considered as a process that suppresses malignant transformation based on multiple indirect lines of evidence showing that inhibition of tumor suppressor genes, such as phosphatase and tensin homolog (PTEN) and p53, can lead to decrease in autophagy [158,159]. The predisposed role of Beclin heterozygosity along with reduction of autophagy in a variety of tumors directly implied that autophagy acts as a suppressive role in tumor progression [160,161]. In a similar fashion, mosaic depletion of ATG5 and conditional knockout of ATG7 in mice resulted in spontaneous formation of benign liver tumors [162]. In addition, Komatsu and colleagues demonstrated that suppression of autophagy, which is accompanied by marked accumulation of p62, a selective autophagy substrate, contributes to the development of hepatocellular carcinoma through persistently activating nuclear factor (erythroid-derived 2)-like factor 2 (Nrf2), a transcriptional factor that activates gene expression of numerous cytoprotective genes [163,164]. In addition to the suppressive role of autophagy in tumorigenesis, mounting evidence suggested that autophagy can promote survival of cancer cells. For example, autophagy process has been shown to be activated in the tumor regions during hypoxia and limited nutrient, thus protecting tumor cells against stress and cell death [165]. Moreover, autophagy is also induced by chemotherapy in anti-cancer treatment, and interference with autophagy can synergistically enhance the efficacy of anti-cancer drugs to kill cancer cells [166,167]. These studies conclude that cancer cells may exploit autophagy pathway to counteract with a variety of cellular stress responses, ensuring cell survival in the progress of tumor development. Along with these findings, it is conceivable that HCV-activated autophagic response may contribute to the tumorigenesis of HCV-associated hepatocellular carcinoma. For example, HCV may pirate autophagic process to benefit virus growth and interfere with the tumor suppressive function of autophagy, finally leading to tumorigenesis of viral-infected cells. In contrast, HCV-induced autophagy could help the chronically-infected cells to circumvent the stresses-induced cell death, such as apoptosis, thus promoting the surveillance of damaged cells and finally resulting in the formation of tumor cells. Apart from its roles in the regulation of lipid metabolism and tumorigenesis, HCV-induced autophagy has recently been reported to participate in the disturbance of glucose homeostasis and the dysregulation of insulin signaling [93], indicating that autophagic response has its contributory role in the HCV-induced insulin resistance. However, the investigation of HCV and its associated alteration in lipid metabolism, the development of hepatocellular carcinoma, and the insulin resistance relies on the *in vivo* HCV infectious model. Hence, it is urgently needed to establish a small animal model that can supports the complete HCV life cycle. Moreover, additional studies are required to understand the detailed mechanism of how HCV activates autophagy and to provide comprehensive knowledge on the physiological significance of autophagic process in HCV-associated diseases.

### 3.3. Apoptosis

HCV has been reported to activate apoptotic cell death via death receptor-triggered extrinsic pathway or mitochondria-mediated intrinsic mechanism [45,104,105,106,107,108,109,110]. On the contrary, certain viral proteins have been shown to inhibit apoptosis in cells [111,112,113,114]. In the cells harboring the replication of HCV viral RNA or complete life cycle of infectious HCV, both extrinsic and intrinsic apoptosis pathways have been respectively demonstrated to be specifically activated [108,115,116,117]. Collectively, these studies support a notion that HCV may differentially regulate apoptosis. Accumulated evidence implies that apoptosis has its potential role in the pathogenesis of chronic liver disease by inducing liver inflammation, liver injury, and liver fibrosis. First, activation of death receptor-mediated apoptosis may stimulate hepatic inflammation and lead to fulminant liver failure by promoting neutrophil extravasation and inducing expression of chemokine [168,169,170,171]. Second, clearance of apoptotic debris through hepatic stellate cells (HSCs)-mediated phagocytosis may stimulate liver fibrogenesis through inducing transform growth factor (TGF)-β and collagen I expressions [172,173]. Third, dysregulation of apoptosis may lead to hepatocarcinogenesis by altering expression of pro-apoptotic molecules and overactivation of anti-apoptotic pathway [174,175,176,177,178,179,180]. Along with the results of these studies, it is conceivable to determine whether viral-altered apoptotic signaling leads to pathogenic change of HCV-associated liver diseases. For example, to investigate whether HCV-induced upregulation of death receptor expression can trigger liver injury and fibrosis via inducing liver inflammation via activating intracellular signaling is needed. On the other hand, it is worthwhile to investigate whether HCV antagonizes the apoptosis pathway to introduce the overacting cell survival signal, thus promoting unregulated cell proliferation and finally leading to development of hepatocellular carcinoma. Nevertheless, these future studies also rely on the establishment of a small animal model that harbors a complete infectious life cycle of HCV and its related pathogenesis. 

### 3.4. Cell Cycle Arrest and DNA Damage, Mitogenic signaling, and PI3K pathway

HCV was shown to alter cell cycle progression by multiple mechanisms. (1) HCV core protein decreases the RB abundance and enhances E2F transactivation of S-phase genes, thus promoting cell proliferation [122]. (2) HCV NS5B protein interacts with RB and targets RB to degradation via an E6AP E3 ligase-dependent pathway, promoting activation of E2F-dependent transcriptional activation [123,124]. (3) HCV infection induces chromosomal polyploidy and impairs mitotic checkpoint by upregulation of Mad2 [126]. Since RB exert its critical function as a tumor suppressor in tightly regulating cell proliferation and apoptosis, it is conceivable that HCV-induced disruption of Rb-E2F-regulatory pathway in the infected cells may promote the uncontrolled hepatocellular proliferation, an initial factor leading to development of liver cancer. Moreover, the chromosomal instability induced by the aberrant G2/M transition and a defect in mitosis in cells upon HCV infection could provide an alternative mechanism to promote HCV-associated hepatocarcinogenesis [126,127]. On the other hand, interference with DNA damage/repair pathway by HCV may make the infected cells sensitized to external stimuli and allow the infected cells to tolerate the massive mutations of genetic information, finally leading to tumorigenesis [128,129,130]. Modulations of MAPK and PI3K signaling pathways have widely been described in a range of tumor cells [131,181]. Deregulation of MAPK signaling by HCV may perturb the control of host cell growth in response to extracellular stimuli [132,133,134,135,136,138,139]. In addition, dysregulation of MAPK pathway by HCV may change the global protein translation, thus affecting the programmed cell growth [136]. On the other hand, activation of PI3K-AKT pathway by HCV has its functional impact on the downstream signaling cascades, such as activation of transcription factors, inhibition of proapoptotic molecules, and activation of oncogene [136,140]. Notably, uncontrolled apoptosis and oncogene activation have been implicated in the development of HCC, suggesting that HCV-altered cellular signaling pathways could be involved in the pathogenesis of HCV-related liver diseases [180,182]. However, deeper investigation into how HCV infection causes cancer will be further needed. Without a small animal model which can support HCV infection of hepatocytes *in vivo* and the entire virus life cycle, it is not easily to answer questions like whether HCV promotes proliferation of liver cell, whether HCV infection interferes with cell cycle checkpoint, and whether HCV infection leads to aberrant mutations in oncogene or tumor suppressor genes. In this regard, an animal model capable of supporting complete HCV life cycle is critically necessary for dissecting how a change in cell cycle progression and DNA damage response affects pathogenesis of HCV-associated diseases. 

## 4. Conclusion and Future Directions

In sum, the interrelationship between host cellular response and HCV-associated pathogenesis of liver diseases has become an attractive topic in the HCV research. Although some mechanistic insights concerning the role of virus-cell interactions have been provided from *in vitro* and *in vivo* models, it is still not possible to precisely identify the exact role of a certain cellular response at a pathogenic stage of HCV-associated disease. Most importantly, supporting evidence from infected patients and *in vivo* small animal model supporting complete HCV life cycle are desperately needed to understand the bona fide physiological and clinical relevance of cellular response in the development of HCV-derived liver diseases. With increasing understanding of how the HCV-induced host stress response causes the pathogenesis of chronic liver diseases, the task of exploration of new strategies for curing HCV infection and for the intervention of HCV-associated liver diseases remains promising and will be possible in the future.
